# The Lytic Activity of Bacteriophage ZCSE9 against *Salmonella enterica* and Its Synergistic Effects with Kanamycin

**DOI:** 10.3390/v15040912

**Published:** 2023-03-31

**Authors:** Abdallah S. Abdelsattar, Mohamed Atef Eita, Zainab K. Hammouda, Shrouk Mohamed Gouda, Toka A. Hakim, Aghapy Yermans Yakoup, Anan Safwat, Ayman El-Shibiny

**Affiliations:** 1 Center for Microbiology and Phage Therapy, Zewail City of Science and Technology, Giza 12578, Egypt; 2 Microbiology and Immunology Department, Faculty of Pharmacy, October University for Modern Sciences and Arts (MSA), Giza 11787, Egypt; 3 Faculty of Environmental Agricultural Sciences, Arish University, Arish 45511, Egypt

**Keywords:** phage therapy, phage-antibiotic synergy, *Salmonella enterica*, bioinformatics analysis, heat map, phage isolation, phage interaction, DNA extraction

## Abstract

*Salmonella*, the causative agent of several diseases in humans and animals, including salmonellosis, septicemia, typhoid fever, and fowl typhoid, poses a serious threat to global public health and food safety. Globally, reports of therapeutic failures are increasing because of the increase in bacterial antibiotic resistance. Thus, this work highlights the combined phage–antibiotic therapy as a promising approach to combating bacterial resistance. In this manner, the phage ZCSE9 was isolated, and the morphology, host infectivity, killing curve, combination with kanamycin, and genome analysis of this phage were all examined. Morphologically, phage ZCSE9 is a siphovirus with a relatively broad host range. In addition, the phage can tolerate high temperatures until 80 °C with one log reduction and a basic environment (pH 11) without a significant decline. Furthermore, the phage prevents bacterial growth in the planktonic state, according to the results of the time-killing curve. Moreover, using the phage at MOI 0.1 with kanamycin against five different *Salmonella* serotypes reduces the required antibiotics to inhibit the growth of the bacteria. Comparative genomics and phylogenetic analysis suggested that phage ZCSE9, along with its close relatives *Salmonella* phages vB_SenS_AG11 and wksl3, belongs to the genus *Jerseyvirus*. In conclusion, phage ZCSE9 and kanamycin form a robust heterologous antibacterial combination that enhances the effectiveness of a phage-only approach for combating *Salmonella*.

## 1. Introduction

*Salmonella* is a type of bacterium that can cause a variety of illnesses in both animals and humans. It is a Gram-negative, rod-shaped, facultatively anaerobic, and motile microorganism known for causing conditions such as salmonellosis, septicemia, typhoid fever, and fowl typhoid [1,2]. *Salmonella bongori* and *Salmonella enterica* (*S. enterica*) comprise the genus, with *S. enterica* divided into six subspecies [3]. *S. enterica*, which has more than 2500 serotypes, is the primary cause of enteric illness [4]. *S. enterica* serotype Typhi causes typhoid fever. Furthermore, it can spread through asymptomatic humans who have previously carried the infection, which is still a major public health issue in developing countries [5]. *Salmonella* is a member of the Enterobacteriaceae family and spreads through contaminated water, food, and the environment. Moreover, it is a commensal microorganism in the intestinal tracts of many animals, including pets, livestock, and poultry [3]. Humans can contract *Salmonella* through various means, such as consuming contaminated food or water or coming into direct contact with infected animals [6]. Furthermore, it can enter the food chain through several routes, including poultry processing sites, animal feed, continuously contaminated livestock environments, vertical transmission, and contaminated hatcheries. Additionally, it can also spread to humans through the fecal–oral route. Once humans contract it, *Salmonella* can lead to salmonellosis, typically developing within 12–72 h and characterized by symptoms such as abdominal cramps, fever, diarrhea, and vomiting. As a result, it is estimated that *Salmonella* causes 1.3 billion cases of gastroenteritis worldwide each year, with three million deaths reported [7]. Additionally, more infections were reported in developing countries, which significantly negatively impacts their economy and health [7,8].

Antibiotics in countries that produce poultry are used to treat salmonellosis and promote growth [9]. To reduce the usage of antibiotics in agriculture and poultry production, several countries have implemented measures to decrease their usage. In 1986, Sweden banned the usage of any antibiotics that promote growth [10]. Then, in 1995 and 1998, Denmark outlawed avoparcin and virginiamycin usage [11]. The European Union (EU) took action in 1997 and 1998 by banning the usage of Avoparcin and other drugs (such as spiramycin, bacitracin, virginiamycin, and tylosin) as growth promoters [12]. For instance, Namibia was one of the first countries in Africa to implement a restriction on the extensive usage of antibiotics in livestock [13]. Before the Food and Drug Administration (FDA) regulated antibiotics at the beginning of 2017, many antimicrobials were given to livestock in the US to promote growth [14]. Unfortunately, the improper use of antibiotics results in the rise and spread of pathogens resistant to antibiotics, making antibiotics useless for the clinical management of infections [15]. According to current trends in antimicrobial resistance (AMR), areas where poultry is produced act as a significant gene repository for AMR [16]. Numerous novel approaches are being researched to offer a long-term solution due to the high mortality, morbidity, and expense of treating diseases brought on by multidrug-resistant (MDR) pathogens [17,18,19,20,21]. Therefore, there is a need to discover alternatives that work in harmony with antibiotics to obtain a synergistic effect, as the antibiotics alone are not enough in many cases [22,23].

Bacteriophages (phages) are bacteria-infecting viruses that can be lytic or lysogenic [24]. Using phages is a promising treatment option for preventing bacterial infections in humans and poultry. Researchers now view phages as a potential alternative to antibiotics due to their powerful bacteriolytic activity, host specificity, self-limiting properties, and ease of genetic manipulation [25]. In addition, phages can survive in various temperatures and pH levels and infect various bacteria. As a result, they are promising candidates for therapeutic use. Numerous studies have documented how effective host-specific phages are at reducing bacterial counts in various foods, including meat, vegetables, eggs, processed foods, and animal skin [26,27,28,29]. However, using phages exclusively frequently results in the rise of phage-resistant bacteria [30]. In addition, recent research revealed the emergence of phage-resistant mutants both in humans and in vitro [31,32,33]. Thus, investigations into the synergistic effects of combining phages and antibiotics are being conducted to prevent the evolution of bacterial resistance to both. Previous studies on phage–antibiotic synergy demonstrated positive outcomes in eradicating bacteria [34,35,36]. The novel virulent phage ZCSE9 was isolated and characterized in this study, and the effects of phage–antibiotic combinations against six species of the human pathogen *S. enterica* were reported. Different concentrations of kanamycin, together with phage ZCSE9, were investigated to evaluate the synergy against these pathogens.

## 2. Materials and Methods

### 2.1. Bacterial Cultures and Preparations

The bacterial strains that were selected for the host range screen and other experiments are listed in Table 1. In addition, *S. enterica* subsp. *enterica* serotype Typhi NCTC 160 was used as a host strain for the isolation and characterization of the phage. The bacterial culture was grown at 37 °C in tryptone soy broth (TSB) (Oxoid, Basingstoke, UK) or xylose lysine deoxycholate (XLD) (Merck, Darmstadt, Germany).

### 2.2. Isolation, Purification, and Amplification of the Phage

One gram of Guava collected from a garden in Al Qalyubia, Egypt, was added to 9 mL of TSB containing *S. Typhi* NCTC 160 as the main host bacteria. After incubation for four hours at 37 °C, the sample was pelleted using centrifugation at low speed (6400× *g*) at 4 °C for 15 min twice, and 1% of chloroform was added before each centrifugation. The process of measuring the phage titer in the whole study was conducted using the double-agar overlay plaque assays [37]. For isolating the phage, spots of the sample after purification with different dilutions were added on the surface of bacteria at the 0.5% top agar. Next, the plates were incubated overnight at 37 °C, and then the single clear plaques were picked using a sterile yellow tip to isolate a pure phage. For the phage propagation, the phage ZCSE9 was mixed with *S. enterica* at OD_600_ ~ 0.3 with MOI ~ 1 and kept for four hours (or until the media of phage and bacteria transferred from turbid to clear). Then, centrifugation, as described previously, was conducted to remove the remaining bacteria. Finally, high-speed centrifugation of 15,300× *g* for one hour at 4 °C to pellet the phage and saline magnesium buffer (SM) (100 mM Magnesium sulfate heptahydrate; 10 mM Sodium chloride; 50 mM Tris (hydroxymethyl)aminomethane hydrochloride and adjusted the pH at 7.4) was used to resuspend the phage pellets followed by filtration using a 0.45 μm syringe filter (Chrom Tech, Apple Valley, MN, USA). The phage supernatant was collected, and the phage stock was stored at 4 °C.

### 2.3. Host Range and Lytic Profile

The sensitivity of 24 clinical *Salmonella* isolates to phage ZCSE9 (2 × 10^9^ PFU/mL) was tested using the drop test. Briefly, 50 µL of log-phase bacteria cultures in 4 mL of 0.5% top agar were solidified on 1.5% TSA agar plates. Ten µL drops of the phage stock were pipetted on the soft agar, and the presence or absence of bacterial lysis was recorded after overnight incubation at 37 °C.

### 2.4. Transmission Electron Microscopy (TEM)

The shape and structure of ZCSE9 were examined using TEM, as mentioned before [38]. First, a sample of 10 µL of the phage at a concentration of 10^9^ PFU/mL was stained with 2.5% uranyl acetate. The sample was then placed on a carbon-coated Cu-grid and left to dry for ten min before being examined using TEM. Images of the stained phage were taken at various magnifications using TEM (1230 JEOL, Tokyo, Japan). Moreover, 100 µL of phage ZCSE9 (10^9^ PFU/mL) was added to 1 mL of overnight bacterial culture and then incubated for one hour, followed by the previous steps of imaging using the same TEM for visualizing the interaction between the phage and the bacteria.

### 2.5. Physicochemical Stability

The effects of environmental factors on the phage, such as temperature and pH, were investigated by incubating 1.5 mL centrifuge tubes containing 100 µL of the phage suspension (10^10^ PFU/mL) for one hour at various temperatures (−20, 4, 37, 50, 60, 70, 80, and 85 °C), to examine the temperature stability of the phage. Another 100 µL of the phage suspension (10^10^ PFU/mL) was incubated in the same buffer solution at various pHs, including pH 2, 3, 4, 7, 10, 11, and 12, for one hour at room temperature to test the pH stability of the phage. The phage titer was assessed using the double-layer plate technique.

### 2.6. Determination of Optimal MOI

The MOI of phage ZCSE9 was determined according to previous work [39] with some modifications. Briefly, 100 µL of *S. enterica* (OD_600_ = 0.4, ~10^8^ CFU/mL) and 100 µL of phage ZCSE9 were added to 900 µL of TSB to phage/bacteria ratios 0.001, 0.01, 0.1, 1, and 10. They were incubated at 37 °C for six hours, and then the phage and bacterial titers were determined by double-layer plating.

### 2.7. One-Step Growth Curve

To evaluate the burst size of phage ZCSE9, a one-step growth curve experiment was performed. Firstly, 10 mL of *S. enterica* culture with titer ~10^8^ CFU/mL was infected by phage ZCSE9 with titer 2 × 10^9^ PFU/mL to a final MOI of 0.1. Then, after ten minutes of incubation at 37 °C with shaking at rpm 90 in the water bath, 100 μL of infected growth was moved to a flask containing 10 mL of TSB, and then 200 μL was transferred from the 10 mL TSB flask into a new flask containing 20 mL of TSB. Briefly, 1 mL aliquots were withdrawn from the 20 mL TSB flask at various time points throughout one hour, and all the aliquots were kept in ice. To determine the phage titers, a tenfold serial dilution and spotting assay were applied in this experiment.

### 2.8. Adsorption Rate Assay

The adsorption rate of ZCSE9 was determined using an adsorption rate assay according to Bagińska et al. [40] with minor modifications. First, *S. enterica* host cells were cultured in TSB until they attained an exponential growth phase. After that, the culture was dispensed into nine sterile microtubes, with each tube containing 1 mL of the culture, to which 10 μL of 10^8^ PFUs/mL ZCSE9 phage suspension was added. The resultant mixed suspensions were incubated at 37 °C for durations of 0, 0.5, 1, 1.5, 2.5, 5, 10, 15, and 20 min. The suspensions were then centrifugated at 15,000× *g* for 1 min to obtain a pellet comprising bacterial cells with attached phages. Finally, the supernatant was collected in sterile microtubes and utilized in a double-layer plate technique to determine the titer of unadsorbed phages.

### 2.9. Time Killing Curve

A time-killing curve assay was carried out to examine the antibacterial effect of phage ZCSE9 against the host strain. First, a day-cultured bacteria with OD_600_ of 0.4 was diluted with TSB to prepare a 5 × 10^6^ CFU/mL bacterial suspension. Next, 180 μL of the bacterial suspension was distributed into 96-well plates. Then, each well was infected with phage (20 μL) at different MOIs (10, 1, 0.1, 0.01, 0.001, and 0.0001) and incubated for five hours at 37 °C. The absorbances at OD_600_ were recorded using a FLUOstar Omega microplate reader (BMG LABTECH, Ortenberg, Germany) at 20 min intervals during the experiment.

### 2.10. Phage DNA Extraction and Genome Sequencing

The DNA extraction of phage ZCSE9 was conducted as described previously [41], with some modifications. Firstly, 1 mL of lysis buffer containing proteinase K (100 µg/mL) was added to 10 mL of stock phage buffer at 56 °C for one hour. Next, 10 mL of phenol/ chloroform/isoamyl alcohol (25:24:1) was added before centrifuging for 10 min at 10,976× *g* at 4 °C. Next, the aqueous layer on top of the chloroform was transferred to another falcon containing an equal volume of isopropanol and 1/10 volume of 3M sodium acetate left overnight at −20 °C. Another centrifugation at 10,976× *g* at 4 °C for 10 min was conducted, followed by discarding the supernatant and collecting the precipitated DNA. Then, the pellet was washed with absolute ethanol and 70% ethanol, and the supernatant ethanol was withdrawn with sterile tips. Finally, the DNA was dissolved in DNAse and RNAse-free water. The DNA concentration was measured with a FLUOstar Omega microplate reader. Next, the DNA was cleaved using the restriction enzyme, SmaI, following the protocols stipulated by the supplier (NEB). The conditions and buffers were selected based on the manufacturer’s recommendations, while the enzymatic incubation was conducted for 1 h, followed by deactivation at 65 °C for 20 min. Subsequently, the cleaved DNA sample was subjected to electrophoresis in a 1.0% (*w*/*v*) agarose gel at 90 V, employing a DNA ladder as a reference marker, and stained with ethidium bromide for visualization. The genome of phage ZCSE9 was sequenced by the Illumina MiSeq platform according to the instructions using the Illumina Nextera tagmentation protocol (Illumina, Cambridge, UK).

### 2.11. Genome Analysis

#### 2.11.1. Assembly and Annotation

The sequence reads were de novo assembled with Unicycler (v0.4.8) on the BV-BRC portal. ProgressiveMauve and Ugene were used for visualization, comparison, and genome orientation according to *Salmonella* phage vB_SenS_AG11 with accession number NC_041991 [42,43]. The assembled genome was annotated using Rapid Annotation using Subsystem Technology Toolkit (RASTtk) pipeline by customizing the annotation to start with “annotate-proteins-phage” followed by “annotate-proteins kmer-v2” [44]. A second round of annotation was performed after RASTtk annotation to confirm assigned functions or to assign functions to proteins with unassigned functions. NCBI BLASTp, HHPred, and InterPro Scan were used for this purpose. ARAGORN v1.2.41 was used to identify tRNA genes [45]. The presence of temperate genetic markers, bacterial virulence, and AMR genes was determined using PHageBACterioPHage LIfstyle Predictor (BACPHLIP v0.9.6) and PhageLeads to evaluate the phage’s potential for therapeutic applications [46,47,48]. The circular genomic map was drawn using CGView on the PROKSEE server [49]. Furthermore, Phage Depolymerase Finder (PhageDPO) was used to detect the genes with putative depolymerase function [50], and DeepTMHMM was used to analyze the topology of the transmembrane domains in the predicted proteins [51].

#### 2.11.2. Phylogenetic Analysis

Genome–genome comparison for ZCSE9 and *Salmonella* phage vB_SenS_AG11 was conducted using the ViPTree server; the latter was also used to construct a circular and a rectangular proteomic tree based on clustering phages at the family level [52]. The output of ViPtree was used to identify phages with the highest tBLASTx scores (*S_G_*) and outgroup phages with the lowest (*S_G_*) scores which were used along with top BLASTn hits from NCBI as input for the virus intergenomic distance calculator (VIRIDIC). The VIRIDIC tool was used to compute pairwise intergenomic similarities between phage ZCSE9 and the selected phages [53].

Furthermore, PhageClouds was used to visualize the relationship between the genome of phage ZCSE9 and other phage sequences on NCBI-GenBank based on their intergenomic distances with a threshold of 0.2 [54].

More phages filtered from the NCBI virus database (filtration criteria was *Jerseyvirus* and complete RefSeq genome) were analyzed using CoreGenes 5.0 to identify terminase genes conserved through the genus and family levels [55]. Terminase, a large subunit (TerL, signature gene), was used to construct a protein-based phylogenetic tree using the phylogeny.fr web-based tool [48,56]. The TerL tree was constructed to verify the assignment of phage ZCSE9 to the genus of the closely related matches according to intergenomic similarity analysis. In addition, the terL tree included outgroups of phages that belong to other genera assigned to class *Caudoviricetes*.

### 2.12. Phage-Antibiotic Synergy

The minimal inhibitory concentrations (MICs) were measured as mentioned by Prashik et al. [57]. After the incubation, the procedure involved checking the difference in Salmonella isolate growth in tryptic soya broth (TSB; Oxoid, Basingstoke, UK). The bacterial isolates were first grown on XLD (Merck, Darmstadt, Germany) plates. Then, colonies were withdrawn and cultured into TSB, which was incubated for four hours at 37 °C. The experiment used kanamycin at different concentrations ranging from 500 to 3.75 μg/mL and phage ZCSE9 at a high titer (10^7^ PFU/mL) as the final concentration to evaluate their antibacterial efficiency at a multiplicity of infection (MOI) of 0.1. A negative control of approximately 100 µL of *S. Typhi* (NCTC 160) was added to a 96-well plate. Then, 5 µL of phage ZCSE9 was added to 100 µL of *S. Typhi* (NCTC 160). Next, 100 µL of the bacterial culture was added to the same volume of kanamycin only. The mixture was adjusted to be diluted two-fold in each well with the final concentration of the antibiotic in the microtiter plates ranging from 3.75 to 500 μg/mL. Then, 100 µL of the bacterial isolate was added to 100 µL of kanamycin and 5 µL of phage ZCSE9 at MOI 0.1. The mixture was adjusted to be diluted two-fold in each well. The clear well with the lowest concentrations of kanamycin only and the kanamycin-phage mixture was identified as the MIC after a 24 h incubation period. From each clear well, as well as the wells containing phage incubated with bacteria and the negative control, 50 µL of TSB was withdrawn and added to 450 µL of fresh liquid media followed by serial dilution, and 10 µL of each dilution was spotted on TSA plates and then incubated at 37 °C overnight. The same steps were repeated using five different bacteria: *S. Typhimurium* (NCTC 13348), *S. Gallinarum* (CMPZC49G.B), *S. Blegdam I* (CMPZC53 sple), *S. Blegdam II* (CMPZC52 int), and *S. Enteritidis* (CMPZC78 Sple). Additionally, a 96-well plate containing *S. enterica* (NCTC 160), *S. Typhimurium* (NCTC 13348), and *S. Gallinarum* (CMPZC49G.B) was incubated for 12 h at 37 °C overnight in a Fluostar Omega multi-mode microplate reader (BMG LABTECH, Ortenberg, Germany) to gather absorbance data, which was used to construct a heatmap showing the percentage reduction in the bacterial population.

## 3. Results and Discussion

### 3.1. Host Range

To determine the role of *Salmonella* in phage infectivity, we used 24 different *Salmonella* bacteria strains to measure the ability of phage ZCSE9 with titer 2 × 10^9^ PFU/mL to infect the bacteria. The lysis activity included 13 bacteria from six serotypes, including Blegdam, Gallinarum, Enteritidis, Kentucky, Typhimurium, and Typhi. However, the phage could not infect *Salmonella* bacteria with serotypes such as Virchom and Nigeria (Table 1).

### 3.2. Morphological Characteristics of ZCSE9

**Figure 1:**
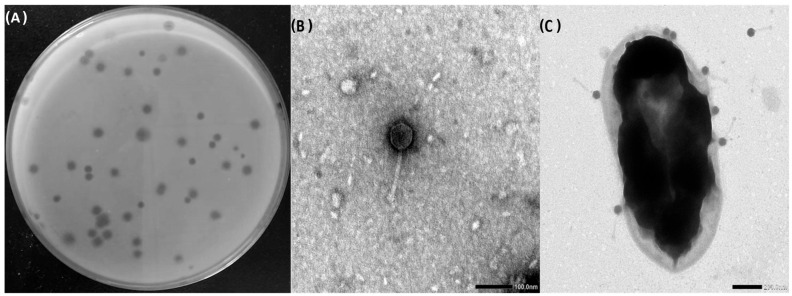
Morphology of phage ZCSE9. (**A**) Plaques of ZCSE9 against *S. Typhi* (NCTC 160), the scale bar 100 nm. (**B**) Phage ZCSE9 TEM micrographs. (**C**) Attachment of ZCSE9 to *S. Typhi* (NCTC 160); scale bar 200 nm.

The plaque morphology of phage ZCSE9 on TSA plates appears to be small, clear, and round, with a halo zone at the periphery of the plaque (Figure 1A). In addition, phage ZCSE9 has an icosahedral head and long, non-contractile tail that is densely packed at the end, resembling a short tail–spikes complex. The phage tail length is 126 nm, and the head is 62 nm in size (Figure 1B). Moreover, TEM shows that virion attachment can involve many phages simultaneously; however, the images show that the phages attach to the bacterial cell wall via their heads while their tails float away (Figure 1C). The phage morphology on the overlay agar shows halo zones, indicating the presence of enzymes that depolymerize exopolysaccharides and biofilm [58]. It was confirmed in different articles previously that the phage from the family *Podoviridae* [59,60] and *Caudovirales* [61] attach to the bacterial surface using its tail. However, we found that phage ZCSE9 binds to the bacterial host with its capsid. This is not the first observation since previous studies reported that phage GMA6 from the same family attached to *Gordonia malaquae* bacteria with its capsid [62]. This phenomenon is not well studied and needs more investigation to confirm the mechanism of interaction between the phage and its bacterial host. We can reveal this phenomenon to the non-contractile tail that makes the attachment with capsid until the tail binds and injects its genetic material, or this bacteria is already infected with the phage previously, and the other phages are unable to bind [63]. However, these hypotheses are not confirmed, and more work is needed to answer the question of the binding using the capsid.

### 3.3. Physicochemical Stability of Phage

**Figure 2:**
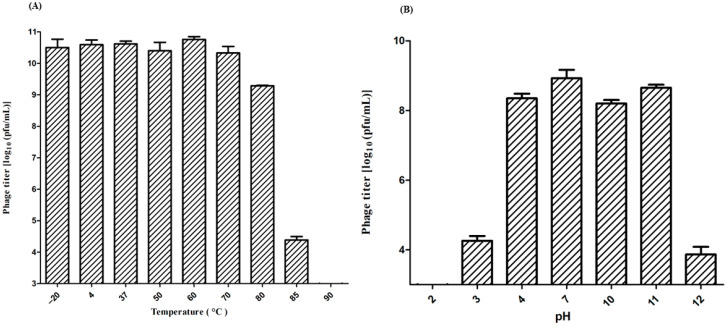
Stability of phage ZCSE9 under different conditions. (**A**) Thermal stability of phage ZCSE9 incubated at different temperatures for one hour. (**B**) pH stability of phage ZCSE9 incubated at different pHs for one hour.

Tolerance to environmental factors was used to investigate the fundamental conditions for further phage applications by determining the variation in plaque-forming unit (PFU) values. Heat stability tests revealed that phage ZCSE9 was completely inactive at 90 °C with a significant reduction in the activity of the phage at 85 °C; however, no significant reduction occurred in the stability of the phage after incubation for one hour at the temperature range of −20 to 80 °C (Figure 2A). In addition, as depicted in (Figure 2B), when phage ZCSE9 was exposed to various pH values, we found that it maintained high activity at a broad pH range (4–11), with the highest titer observed at pH 7. However, the activity sharply decreased in both pH 3 and 12 and was inactive in pH 2.

The physiological stability of phages is crucial because pH and temperature conditions will particularly influence the stability and killing activity of the phage [64]. Moreover, the suitable applications of the phage may be determined based on their stability and activity. Additionally, any antibacterial agent’s capacity to withstand acidic pH conditions is a key requirement. In this study, the phage became completely inactive after exposure to low pH, possibly due to protein denaturation in the virion at pH 2 [65]. However, ZCSE9 can be encapsulated in beads to tolerate the pH levels of the gastrointestinal tract, where *Salmonella* serotypes are typically colonized, to be used as a therapeutic [66,67,68]. The physiological conditions of the human large and small intestines are consistent with these pH values.

The phage can be stored safely at temperatures lower than 0 °C for a short time. However, previous works showed a reduction in phage titer at temperatures below the freezing point due to crystal formation with a recommendation to use glycerol [69]. Thus, we recommend further experiments to measure stability when frozen for a longer time. Moreover, the thermal stability of ZCSE9 against different temperatures from −20 °C to 85 °C was reported (Figure 2A). The most interesting part of these results is that this phage tolerated the temperature of 80 °C for one hour with around one-log reduction. Previously, one phage was isolated and characterized as a thermotolerant phage, phage 936, which tolerated the temperature of 80 °C for 5 min with around half-log reduction, and phage P1532 tolerated 90 °C for 20 min or at 97 °C for 5 min with a high reduction [70].

### 3.4. Optimal MOI, One-Step Growth Curve, and Adsorption Rate Assay

**Figure 3:**
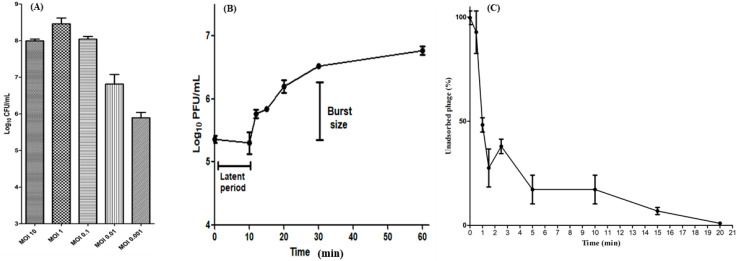
The activity of ZCSE9 against the host bacteria. (**A**) The remained bacteria after six hours by treating them with different MOIs. (**B**) The one-step growth curve of the phage. (**C**) The adsorption curve demonstrates the adsorption percentage over time as measured by the titer of phages remaining in the supernatant.

In this experiment, *S. Typhi* (8 × 10^5^ CFU/mL) was infected at different MOIs and the results showed that the MOI of 0.001 was, under these growth conditions, the most optimal to obtain the highest phage titer (4 × 10^10^ PFU/mL). After that, the phage with MOI 0.01 has activity lower than MOI 0.001 and better than 0.1, 1, and 10 (Figure 3A).

A one-step growth assay at MOI 0.1 was carried out to demonstrate the lysis capabilities of phage ZCSE9 against *S. Typhi* and to estimate the latent period (the time between phage adsorption and release) in addition to burst size (the number of released virions per single bacteria) of phage ZCSE9. The curve displayed that the latent period was around 10 min; in addition, there was 10 min of preparation, making the process from adsorption to release 20 min. Moreover, the burst size was about 20 phages per single host bacterial cell (Figure 3B).

The results of the adsorption rate assay were represented in a line graph in Figure 3C), which illustrates the percentage of the unadsorbed phage in a specific time by the previously identified host. The experiments showed that the time needed by *S. enterica* to adsorb the largest number of phages is almost 20 min after presenting the phage with its host. Moreover, the results also show that the phage adsorbs very rapidly, as 70% had adsorbed in 2 min.

Based on the results of optimal MOI, we found that a lower MOI (0.001) is more efficient after six hours as it produces a higher number of phages and then kills its host efficiently. Similar results were reported with *Salmonella* phage SP76, which has an optimal MOI of 0.0001 [71]. However, the outcome of this experiment depends on the starting conditions of the experiment. When the total incubation time is selected to be six hours, there is a risk that the bacteria will reach the stationary phase before that. Thus, we recommend further work to investigate the optimal MOIs in lower starting CFU/mL. The process of attachment is necessary to recognize the bacterial host within a few minutes as around 80% of *Salmonella* phages T156 were attached to the bacterial cells within 10 min [72]. This process has many factors controlling the dynamic of the phage. Here, the results of the adsorption rate are compatible with the one-step growth curve, as the phage spent 20 min in the adsorption process, and then the new progenies started to be produced. Moreover, mixing between the bacteria and the phage was used to ensure the maximum number of phages were in attachment with the bacteria with a limitation of free phages.

### 3.5. Lytic Activity

**Figure 4:**
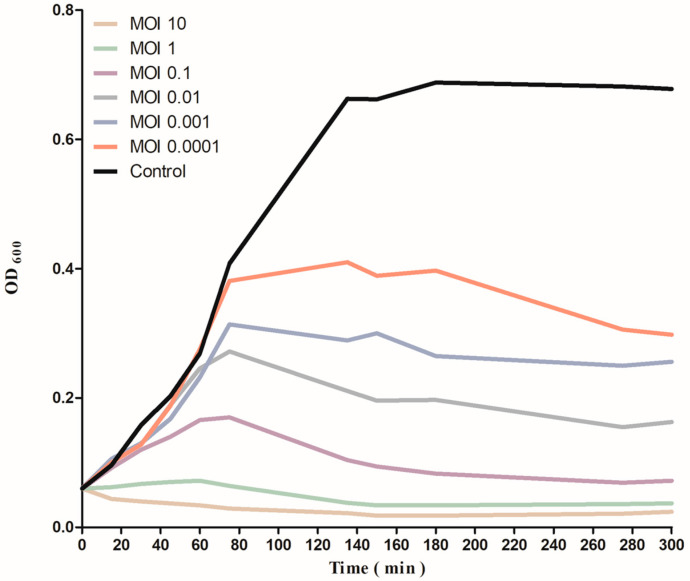
Reduction in *S. Typhi* (NCTC 160) growth by phage ZCSE9 at different MOIs.

At various MOIs (10, 1, 0.1, 0.01, 0.001, and 0.0001), the bactericidal effect of phage ZCSE9 on *S. Typhi* was examined in vitro (Figure 4). After 75 min of the infection period, the bacterial turbidities at OD_600_ were 0.409 for bacterial control (the bacteria that had not been exposed to the phage), 0.38 for MOI 0.0001, 0.31 for MOI 0.001, 0.27 for MOI 0.01, 0.17 for MOI 0.1, 0.06 for MOI 1, and 0.03 for MOI 10. However, the OD_600_ for bacterial control increased significantly from 0.381 to 0.663, and at 180 min, the OD_600_ was 0.688 and then nearly the same for the next 120 min without declining. In contrast, the bacterial growth in all MOIs of ZCSE9 infected cultures declined to display notable bacterial killing activity and hydrolysis.

The bactericidal activity of the phage ZCSE9 was increased by increasing the MOI from 0.0001 to 10. This is not surprising as many previous analyses found that the phage reduces its host more efficiently when used with high MOIs [73,74]. The spectrophotometer provides data supporting bacterial reduction using different antibacterial agents at OD_600_. However, such results can only be measured at a high concentration that gives turbidity using spectroscopy without information about the phage count [75]. Thus, other methods were used to solve the OD problems, such as the spotting technique, which takes a long time but is valid and accurate for all used concentrations [76]. The reason behind the high activity at high MOIs returns to the high chance of a phage finding its host in the solution. However, this is not a problem as the phage is the only antibacterial agent to replicate using a bacterial host with time [77]. In this study, after a long time of using low concentrations of bacteria, the low MOI kills the bacteria better than the high MOI. However, in the turbidity assay, the phage with a high MOI could find and kill the bacteria faster than with a low MOI.

### 3.6. Genomic Characterization of Phage ZCSE9

#### 3.6.1. Analysis and Annotation of the Genome

**Figure 5:**
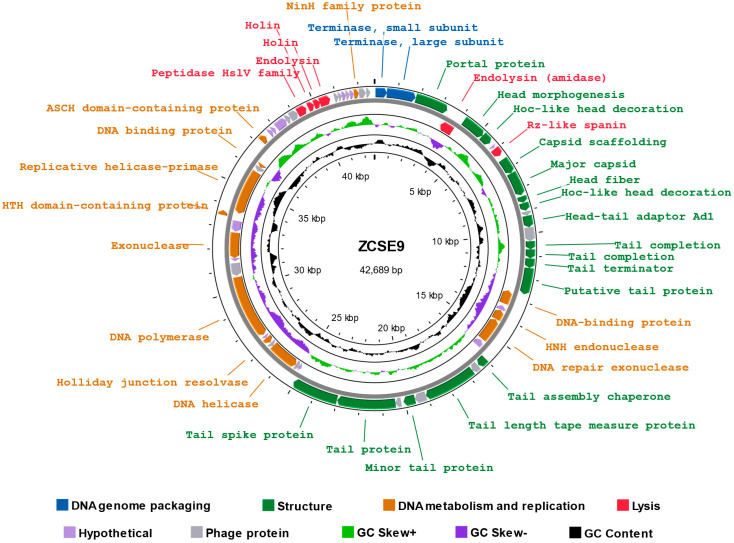
Genomic map of ZCSE9, created with PROKSEE software. The color coding represents the coding sequences (CDS) according to different categories of the predicted function: DNA genome packaging (blue); structure (green); DNA metabolism and replication (orange); lysis (red); phage and hypothetical proteins (shades of grey); GC content skew (black); GC skew (green and purple).

The reads were assembled into one contig, with a double-stranded DNA genome length of 42,689 bp and an overall GC content of 49.7% (Accession: OP793478.2). The predicted open reading frames (ORF) in the complete genome of ZCSE9 phage were 65, 37 of which were assigned to various proteins functions, including DNA genome packaging (terminase small subunit and terminase large subunit), structure proteins (portal protein, head fiber, head morphogenesis, major capsid protein, capsid scaffolding, tail completion, tail terminator-like protein, and tail assembly chaperone), DNA metabolism and replication proteins (DNA polymerase, helicase, HNH endonuclease, and Holliday junction resolvase), and lysis proteins (holin, endolysin, spanin, and peptidase) (Table S1) with no detected tRNA gene. Moreover, there were no detectable lysogenic phage-related markers in phage ZCSE9, such as transposases or integrase. The genomic map illustrates functional genes and their position on the genome (Figure 5). Phage ZCSE9 has a virulent lifestyle as predicted by BACPHLIP with 100% probability. Similarly, PhageLeads could not detect any predicted temperate lifestyle genes, and no antimicrobial resistance or virulence genes were found either. The PhageDPO tool detected depolymerase activity in the tailspike protein (ORF 19 and 32) by both SVM and ANN models with 100% prediction (Table S2). Moreover, genes encoding putative lysis-associated proteins, including two putative holins, were identified where DeepTMHMM predicted two transmembrane topologies of class I and class II holins (Figure S1). The full data about the genome were released on NCBI under Acc. No. of OP793478.

#### 3.6.2. Phylogenetic Analysis

**Figure 6:**
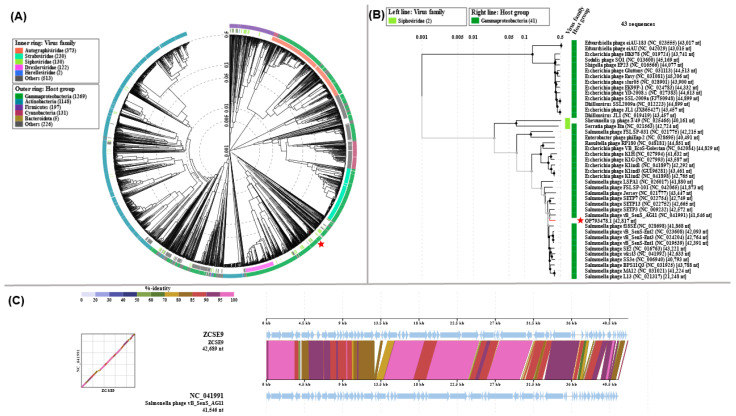
Proteomic phylogeny and whole-genome alignment on ViPTree. (**A**) Circular proteomic tree of ZCSE9 and related RefSeq phage genomes on NCBI. (**B**) Rectangular proteomic tree of top matches to phage ZCSE9. In both trees, phage ZCSE9 is highlighted with a red star. (**C**) Whole-genome alignment of phage ZCSE9 with the closest match *Salmonella* phage vB_SenS_AG11.

The phage ZCSE9 genome was compared to reference phage genomes using ViPTree, which engendered a circular proteomic tree in which phage ZCSE9 clustered among unclassified siphoviruses with a bacterial host of the class Gammaproteobacteria (Figure 6A). Phages with top *S_G_* scores were used to generate a more detailed rectangular tree, in which phage ZCSE9 is clustered with members of the genus *Jerseyvirus* (Figure 6B). Moreover, the whole genome alignment of phage ZCSE9 and its top match *Salmonella* phage vB_SenS_AG11 (NC_041991) is shown in Figure 6C.

**Figure 7:**
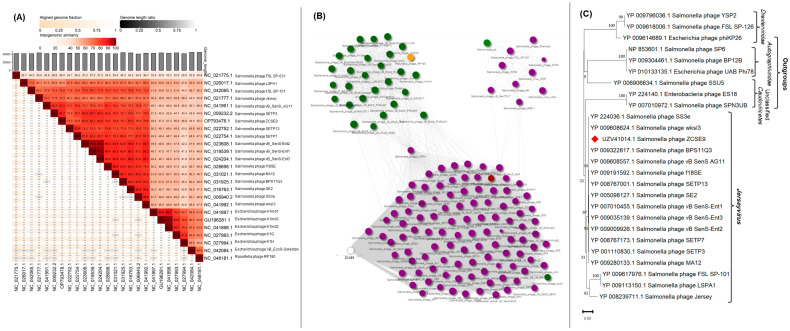
Intergenomic and signature protein-based phylogenetic analysis. (**A**) VIRDIC heatmap for ZCSE9 and closely related phage genomes based on BLASTn top hits. (**B**) PhageCloud analysis of ZCSE9 phage with phage genomes on NCBI-GenBank at a distance threshold of ≤0.2. (**C**) The terL-based phylogenetic tree represents the evolutionary relationship of the terminase large subunit of the ZCSE9 phage to their homologous proteins in other phages and the outgroup. The red diamond in denotes the location of the terminase large subunit of the ZCSE9 phage.

Intergenomic analysis by VIRDIC generated 37 clusters at the species level and 14 clusters at the genus level. VIRDIC grouped the ZCSE9 phage with other unclassified siphoviruses belonging to the same genus, *Jerseyvirus* (Figure 7A, Table S3). The phage cloud computed the distance relationship between phage ZCSE9 and deposited phage genomes on NCBI-GenBank, in which 144 phage genomes were connected to phage ZCSE9 based on a distance threshold ≤ 0.2 (Figure 7B). The phage cloud grouped phage ZCSE9 with phages belonging to the genus *Jerseyvirus.* CoreGene 0.5 identified the terminase large subunit as an ortholog among RefSeq phage genomes classified under the genus of *Jerseyvirus*. Subsequently, a TerL protein-based phylogenetic tree was constructed, which supported the classification of phage ZCSE9 as a new species in the genus *Jerseyvirus* (Figure 7C).

In this study, phage ZCSE9 has properties similar to *Salmonella* phage fmb-p1, including tolerance to high temperatures reaching 90 °C for one hour and survival in harsh basic environments [78]. Bacteriophages are a promising alternative to antibiotics in the struggle against the emergence and spread of bacterial resistance; however, the safety of phage therapy is often questioned [79]. Thanks to the advanced technologies in sequencing and web-based analysis tools, we can easily perform genomic analysis and reveal answers to challenging questions [48]. Genomic analysis of the phage ZCSE9 did not find genes for bacterial virulence, antibiotic resistance, or temperate markers, which supports the phage’s safety.

We could not use bioinformatics tools to determine the genome termini because the library was prepared using the Nextera tagmentation protocol. Methods such as Nextera, which rely on transposases to ligate the adapters, should not yield data acceptable for PhageTerm and similar techniques [80]. Moreover, the putative tailspike endorhamnosidase, identified by PhageDPO and the BLASTp, demonstrates the phage’s ability to cleave the polysaccharide in capsular or biofilm form, which subsequently facilitates the phage invasion and attachment to bacterial cells [81]. Furthermore, the point mutations in the tailspike can expand the host range if it is monovalent [82]. DeepTMHMM identified genes encoding putative lysis-associated proteins, including two putative holins; their topology agreed with class I and II holins [83].

### 3.7. Synergistic Activity of Phage ZCSE9 and Kanamycin

Because antibiotics and phages are essentially different therapeutics, research into such combinatorial therapies is critical. Here, we tested the effectiveness of kanamycin in combination with the lytic phage ZCSE9 against five different *Salmonella* serotypes.

The activity of kanamycin against the six different *Salmonella* bacteria was investigated by an MIC test. The MIC of kanamycin was 15 μg/mL against *S. Typhi* and *S. Typhimurium*, 62 μg/mL against *S. Gallinarum* and *S. Blegdam I*, and 30 μg/mL against *S. Blegdam II* and *S. Enteritidis*. However, the MIC of kanamycin in combination with phage ZCSE9 was 7.5 μg/mL against *S. Typhi* and *S. Typhimurium* and 15 μg/mL against *S. Gallinarum*, *S. Blegdam I*, *S. Blegdam II*, and *S. Enteritidis* as shown in (Table 2).

**Figure 8:**
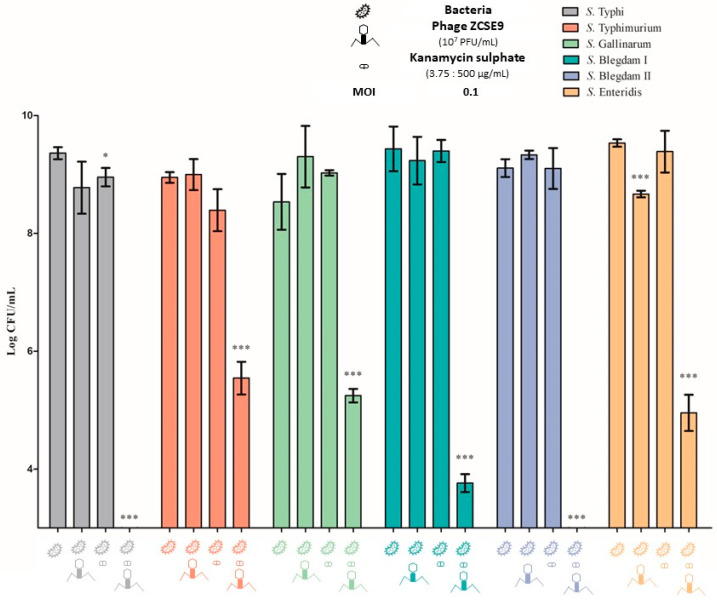
Overnight phage ZCSE9–kanamycin synergy quantification against *S. Typhi* (NCTC 160), *S. Typhimurium* (NCTC 13348), *S. Gallinarum* (CMPZC49G.B), *S. Blegdam I* (CMPZC53 sple), *S. Blegdam II* (CMPZC52 int), and *S. Enteritidis* (CMPZC78 Sple) that was treated with kanamycin concentrations of 7.5, 7.5, 15, 15, 15, and 15 µg/mL, respectively. The ZCSE9 in this experiment was at an MOI of 0.1. The single asterisk denotes significant differences at *p* < 0.05, and the triple asterisk at *p* < 0.001.

Furthermore, the effects of phage ZCSE9 and sublethal concentrations of the kanamycin on *Salmonella* isolates were studied by calculating the viable cell concentration after 24 h of exposure to phage–antibiotic combinations, as shown in Figure 8. The bacteria in the controls without phages and antibiotics reached 8.5–9.5 log CFU/mL, and no significant reduction was observed in the presence of the phage or antibiotic alone. In contrast, a highly significant growth inhibition was detected with the antibiotic–phage combination, whereas when kanamycin was used in combination with phage ZCSE9, the number of bacteria was below the detection limit in the case of *S. Typhi* and *S. Blegdam II*, indicating a significant elimination *p* < 0.001 of bacterial cells. Moreover, the number of bacteria was notably reduced for *S. Typhimurium*, *S. Gallinarum*, *S. Blegdam I*, and *S. Enteritidis* to be 5.5 log CFU/mL, 5.2 log CFU/mL, 3.7 log CFU/mL, and 4.9 log CFU/mL, respectively.

The results from the antibiotic–phage combination confirm a synergistic interaction between kanamycin with the ZCSE9 phage. This finding is particularly noteworthy because it suggests that this combination may be an effective treatment strategy for bacterial infections. Several works have agreed on the efficiency of using phages in combination with traditional antibiotics for treating infections caused by various bacterial pathogens [36,81,82,83]. One key mechanism by which the ZCSE9 phage may enhance the effectiveness of kanamycin is by stressing the bacteria with a sub-lethal dose of the antibiotic. This allows the bacteria to become more lysed using the phage with low and high titers [84]. This is an important consideration because it means that lower dosages of both kanamycin and the ZCSE9 phage may be needed to achieve the desired therapeutic effect. This is particularly beneficial from a pharmacokinetic perspective, as high dosages of the ZCSE9 phage may increase the likelihood that the immune system will recognize and eliminate them from the human or animal body [85].

**Figure 9:**
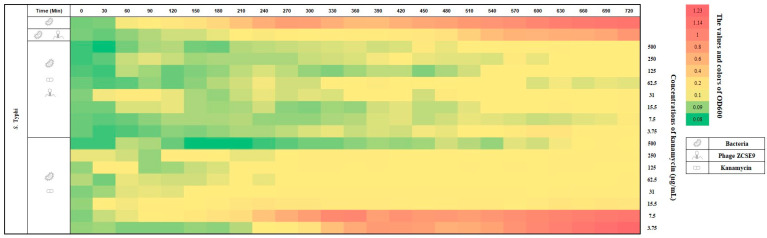
Heat plot showing the synergy of phage ZCSE9 and kanamycin against *S. Typhi* (NCTC 160). The bacterial growth at OD_600_ was represented with colors and numbers in the right vertical bar; red is the highest growth, and green is the lowest.

**Figure 10:**
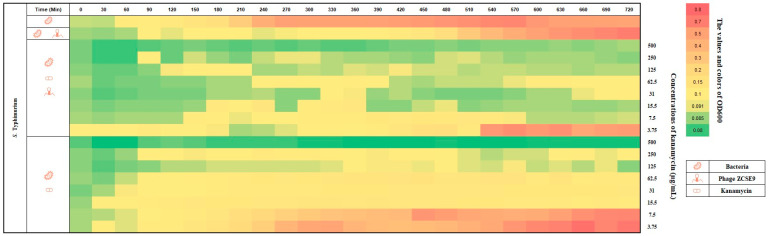
Heat plot for the synergy of phage ZCSE9 and kanamycin against *S. Typhimurium* (NCTC 13348). The bacterial growth at OD_600_ was represented with colors and numbers in the right vertical bar; red is the highest growth, and green is the lowest.

**Figure 11:**
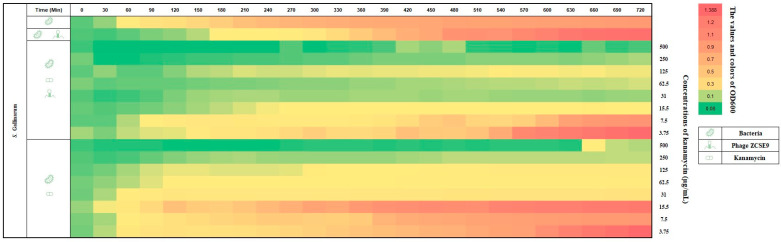
Heat plot corresponding to the synergy of phage ZCSE9 and kanamycin against *S. Gallinarum* (CMPZC49G.B). The bacterial growth at OD_600_ was represented with colors and numbers in the right vertical bar; red is the highest growth, and green is the lowest.

The obtained results in the three heatmaps (Figure 9, Figure 10 and Figure 11) are consistent with the MIC and spotting presented results. For example, in Figure 9, the absorbance of *S. Typhi* (control group) increased from OD_600_ 0.08 (green) to 1.14 (red) in 720 min (12 h). However, when using the phage alone, the absorbance increased from 0.08 to 0.84. In addition, the bacteria in the last rows (0.5× and 0.25× MIC of kanamycin, 7.5 and 3.75 μg/mL, respectively) grew to be similar to those without treatment after the end of the experiment. However, the heat plot was nearly greenish when the phage with an MOI of 0.1 was used with kanamycin. In addition, the bacterial growth increased at concentrations of kanamycin below the MICs (0.25× MIC, 3.75 μg/mL) with the phage with an MOI of 0.1, and the plot became yellow (OD_600_ of 0.1) after 540 min.

Compared to using kanamycin alone, it is easily noticed that after 270 min, the plot became yellow (OD_600_ of 0.1–0.17), and the last two rows (0.5× and 0.25× MIC of kanamycin, 7.5 and 3.75 μg/mL, respectively) became pinkish and then red (OD_600_ of 0.4–1.23). Almost the same result was obtained against *S. Typhimurium* without treatment, as shown in Figure 10. However, the row (0.5× MIC kanamycin, 7.5 μg/mL, with the phage with an MOI of 0.1) remained green (OD_600_ of 0.092–0.089) after 540 min. In addition, the second row shows the activity of the phage alone with an MOI of 0.1 in delaying the growth of *S. Typhimurium*. Therefore, it is necessary to combine with antibiotics to enhance its activity.

In Figure 11, where *S. Gallinarum* was tested against kanamycin and phage ZCSE9 as previously discussed when kanamycin was used with a phage, the results were better, as the row turned red (OD_600_ of 1.245) after 720 min when the 0.25× MIC of kanamycin (15 μg/mL). In contrast, the row was yellow (OD_600_ of 0.118) after the same time when the same concentration was combined with the phage with an MOI of 0.1.

Optimizing the combination of phage therapy and kanamycin could be a promising approach for reducing both dosages and limiting inflammatory immune responses. This is especially important because the overprescription and overuse of antibiotics and other antimicrobial agents can lead to negative side effects and the development of resistance [86]. By using a combination of phage therapy and kanamycin, it may be possible to achieve the same therapeutic effect while minimizing these risks. Moreover, using phages combined with antibiotics may help reduce the selective pressure for antibiotic resistance development, thus preserving the effectiveness of traditional antimicrobials [87,88,89]. We recommend further studies that combine the phage with lower and higher MOIs with different concentrations of antibiotics other than kanamycin to determine the possible differences.

These results suggest that the synergistic effect after using kanamycin and the ZCSE9 could be a promising approach for treating bacterial infections. However, more investigation is necessary to fully comprehend the mechanisms underlying this interaction and maximize its application in clinical settings.

## 4. Conclusions

Our study isolated the broad-spectrum phage ZCSE9, which exhibits a potent lytic effect on *S. enterica* NCTC 160 from guava. It displayed short latent periods as well as basic pH and thermal tolerances. Furthermore, it demonstrated synergistic antibacterial activity against five *Salmonella* serotypes when ZCSE9 and kanamycin were combined. Adding a phage may reduce the antibiotic concentration required to treat *Salmonella* infections. This suggests implementing a lower dosage regimen to help avoid the side effects frequently associated with administering high antibiotic doses. Further work is needed to assure the applicability of combining the phage with antibiotics for therapeutic purposes.

## Figures and Tables

**Table 1:** Lytic activity of phage ZCSE9 against *Salmonella enterica*.

ID	*Salmonella* Bacteria	The Bacteriolytic Activity of ZCSE9
CMPZC52 int	*Salmonella enterica* Blegdam II	+
CMPZC49G.B	*Salmonella enterica* Gallinarum	+
CMPZC78 Sple	*Salmonella enterica* Enteritidis	+
CMPZC18	*Salmonella enterica* spp.	+
CMPZC77 liv	*Salmonella enterica* Kentucky	+
CMPZC53 sple	*Salmonella enterica* Blegdam I	+
CMPZC85 liv	*Salmonella enterica* Virchom	−
CMPZC29 int	*Salmonella enterica* Enteritidis II	+
CMPZC49 liv	*Salmonella enterica* Blegdam III	+
CMPZC52 liv (15)	*Salmonella enterica* spp.	+
CMPZC2	*Salmonella enterica* Kentucky II	−
CMPZC3	*Salmonella enterica* spp.	−
CMPZC7 L	*Salmonella enterica* Typhimurium II	−
CMPZC77 int	*Salmonella enterica* Kentucky III	−
CMPZC100 Sple	*Salmonella enterica* Nigeria	−
CMPZC84 G.B	*Salmonella enterica* spp.	−
CMPZC52 Sple (7)	*Salmonella enterica* Blegdam IV	−
CMPZC 77 liv	*Salmonella enterica* Kentucky IV	+
CMPZC20	*Salmonella enterica* Typhi II	−
CMPZC33	*Salmonella enterica* Typhimurium III	−
CMPZC34	*Salmonella enterica* spp.	+
CMPZC37	*Salmonella enterica* spp.	−
NCTC 13348	*Salmonella enterica* Typhimurium	+
NCTC 160	*Salmonella enterica* Typhi	+

**Table 2:** The MIC of different bacteria against kanamycin alone and with phages.

	Bacteria	ID	Kanamycin Concentration (µg/mL)							
			500	250	125	62	30	15	7.5	3.75
Kanamycin	*S. Typhi*	NCTC 160						√		
	*S. Typhimurium*	NCTC 13348						√		
	*S. Gallinarum*	CMPZC49G.B				√				
	*S. Blegdam I*	CMPZC53 sple				√				
	*S. Blegdam II*	CMPZC52 int					√			
	*S. Enteritidis*	CMPZC78 Sple					√			
Phage MOI 0.1 + Kanamycin	*S. Typhi*	NCTC 160							√	
	*S. Typhimurium*	NCTC 13348							√	
	*S. Gallinarum*	CMPZC49G.B						√		
	*S. Blegdam I*	CMPZC53 sple						√		
	*S. Blegdam II*	CMPZC52 int						√		
	*S. Typhi*	CMPZC78 Sple						√		

## Data Availability

Not applicable.

## Supplementary Materials

The following supporting information can be downloaded at: https://www.mdpi.com/article/10.3390/v15040912/s1, Figure S1: Predicted class I and class II holins using DeepTMHMM; Table S1: Annotated CDS of phage ZCSE9; Table S2: Phage depolymerase finder results for ANN and SVM models; Table S3: VIRIDIC clustering results.
