# Supplementary material for: The Lytic Activity of Bacteriophage ZCSE9 against Salmonella enterica and Its Synergistic Effects with Kanamycin
Source: Viruses. 2023 Mar 31;15(4):912. doi: 10.3390/v15040912 (PMC10142335; doi:10.3390/v15040912)
Supplement: Supplementary file 1 [file viruses-15-00912-s001.zip › viruses-2174575-supplementary.pptx]

## Slide 1
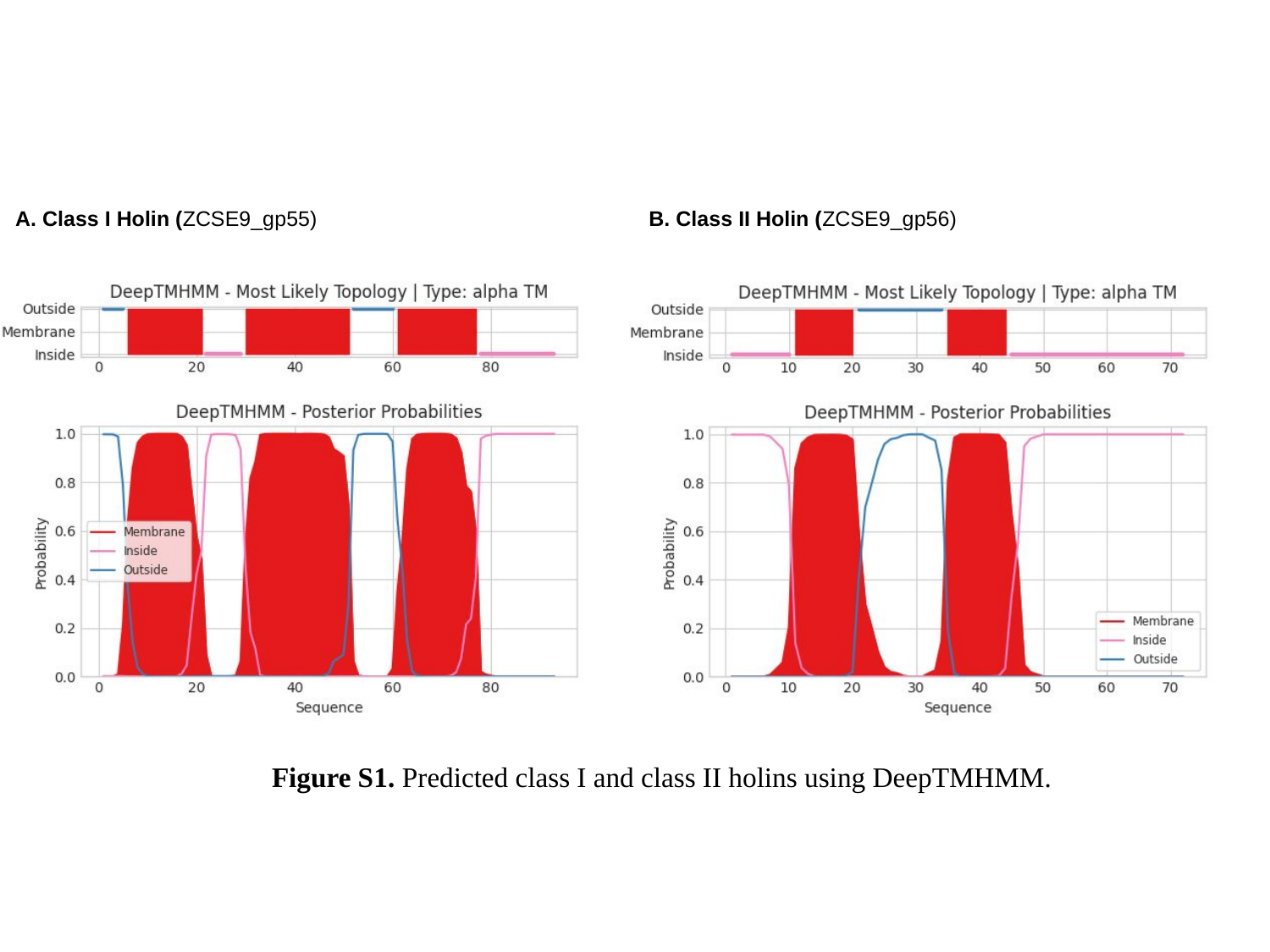

B. Class II Holin (ZCSE9_gp56)
A. Class I Holin (ZCSE9_gp55)
Figure S1. Predicted class I and class II holins using DeepTMHMM.

## Slide 2
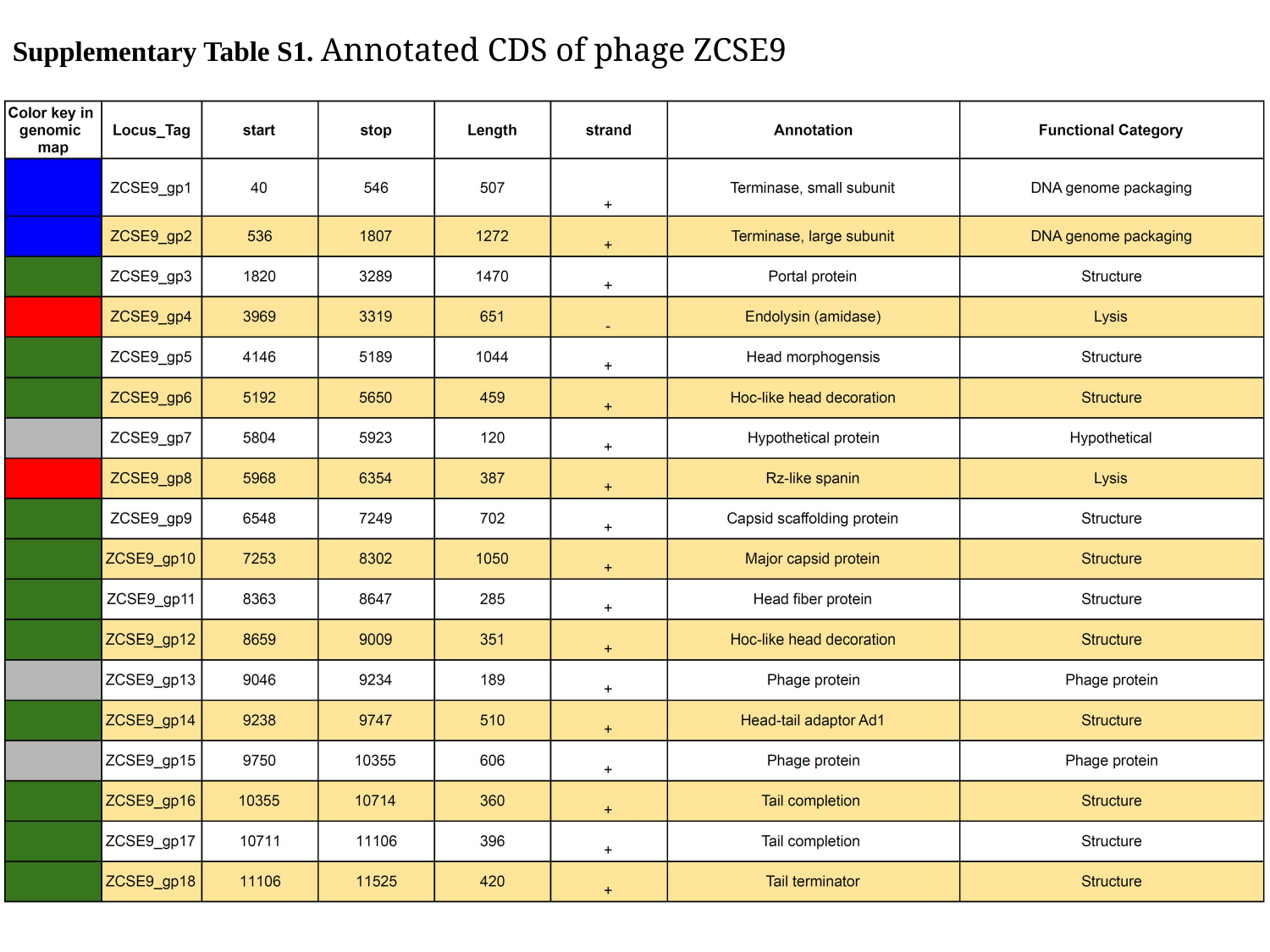

Supplementary Table S1. Annotated CDS of phage ZCSE9

## Slide 3
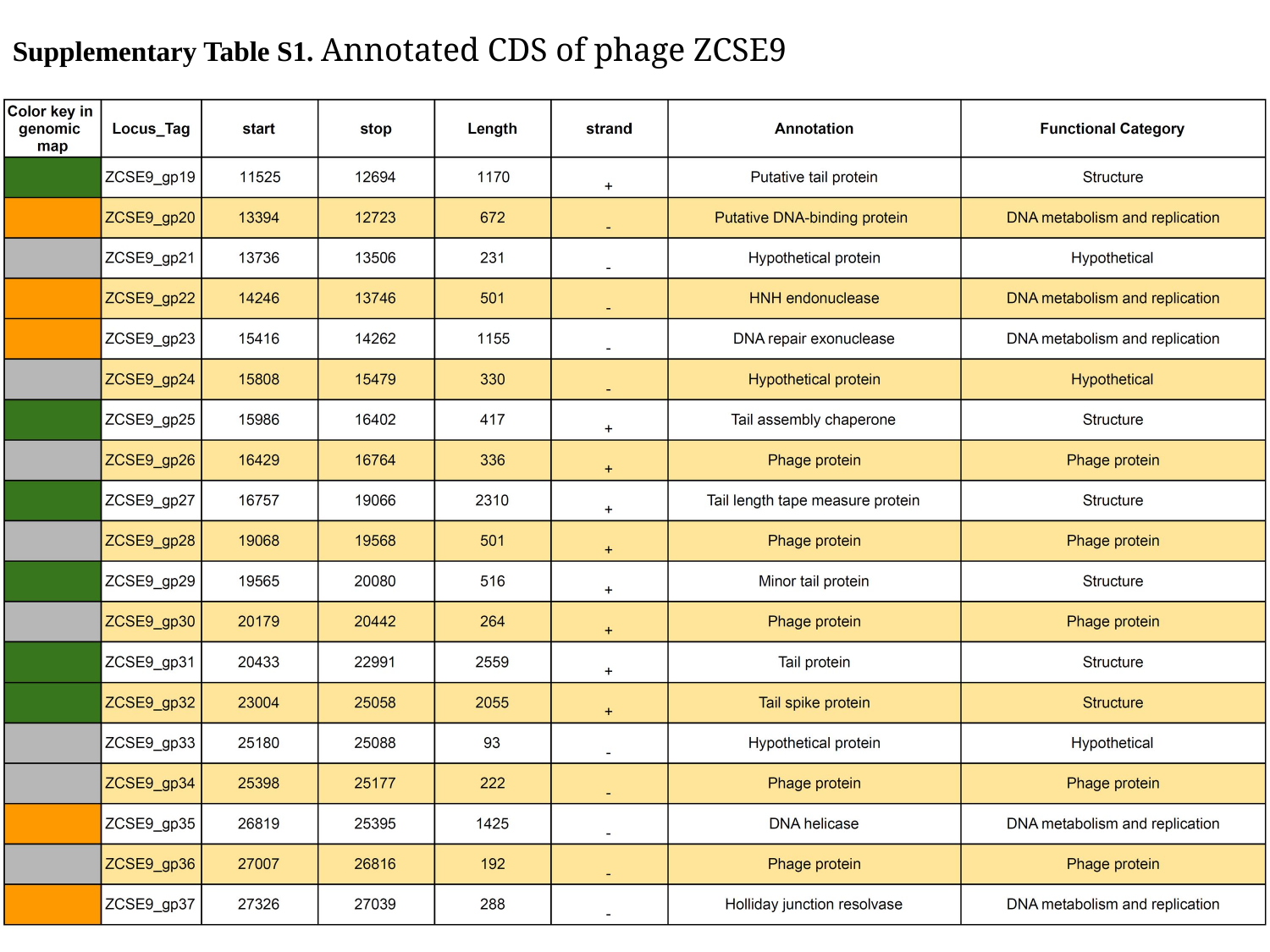

Supplementary Table S1. Annotated CDS of phage ZCSE9

## Slide 4
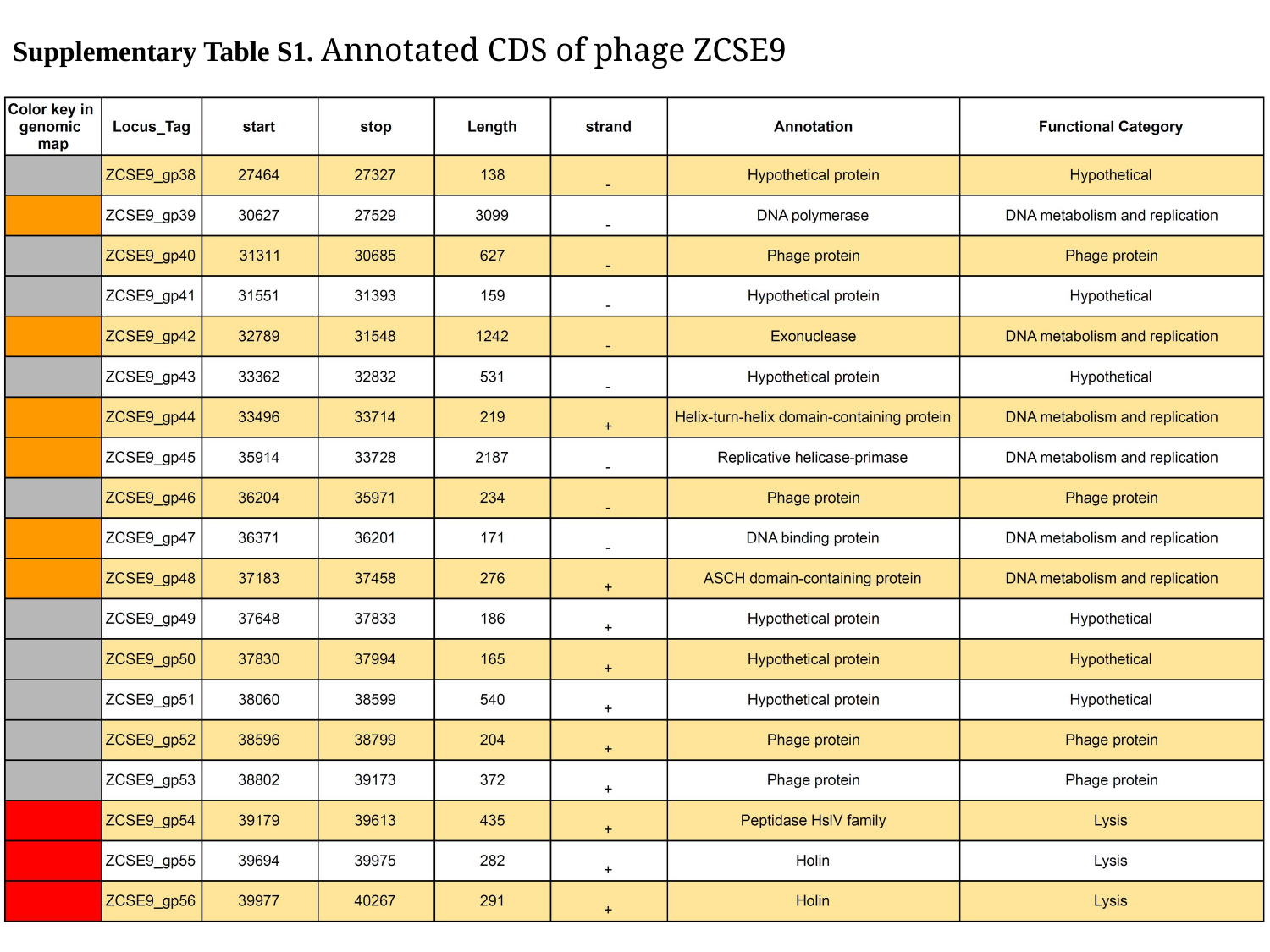

Supplementary Table S1. Annotated CDS of phage ZCSE9

## Slide 5
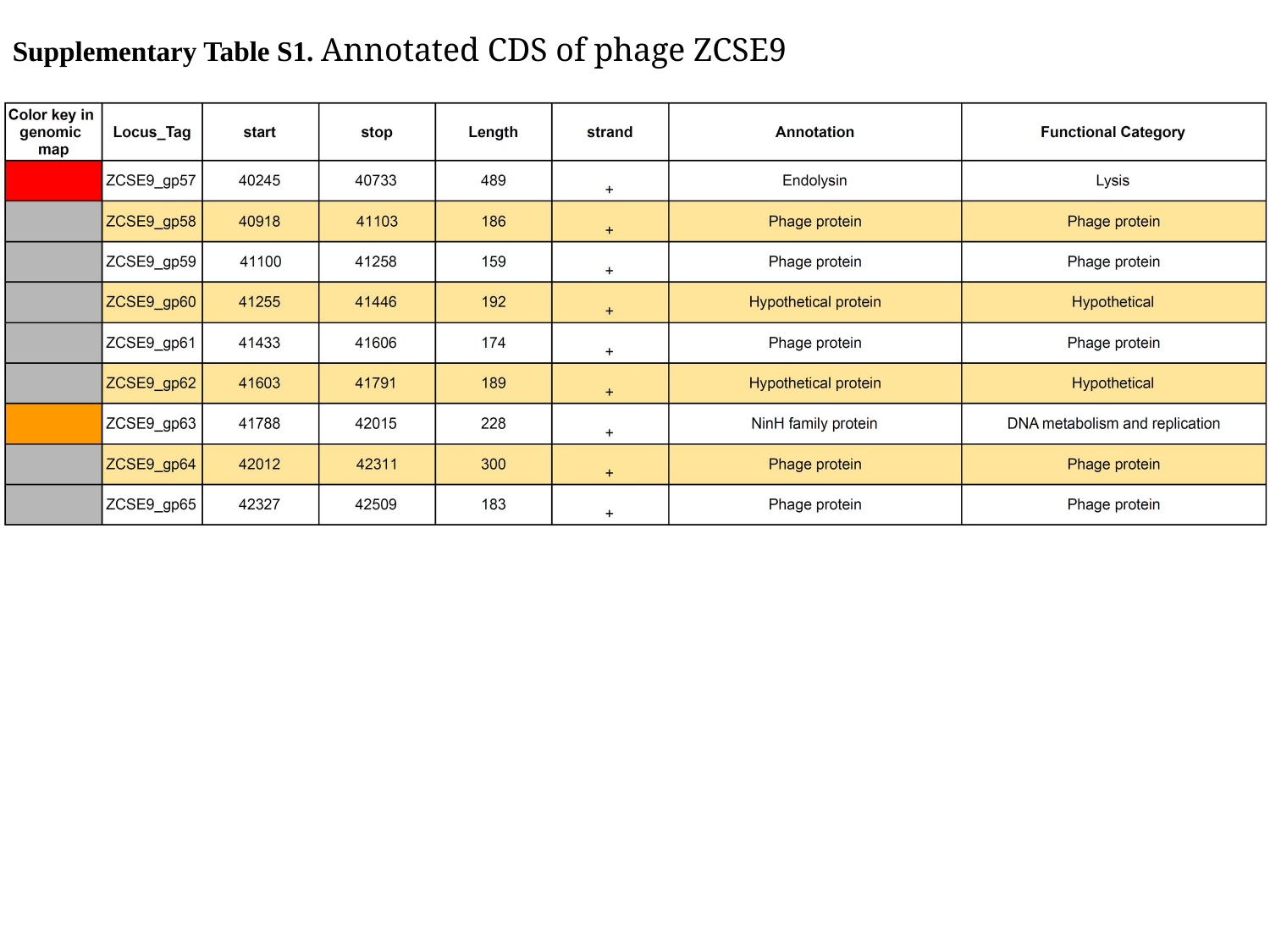

Supplementary Table S1. Annotated CDS of phage ZCSE9

## Slide 6
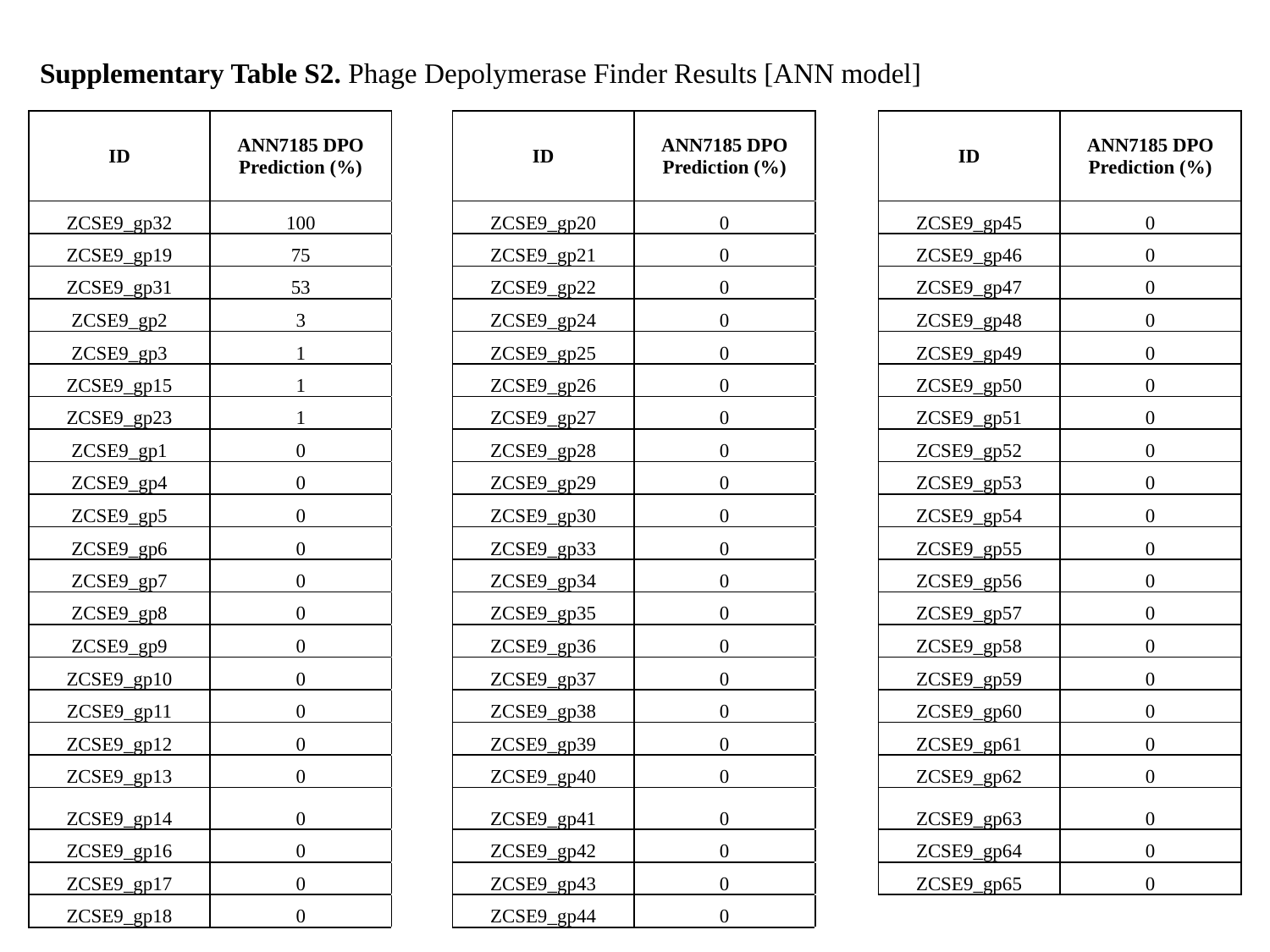

| | | | | | | | |
| --- | --- | --- | --- | --- | --- | --- | --- |
| ID | ANN7185 DPO Prediction (%) | | ID | ANN7185 DPO Prediction (%) | | ID | ANN7185 DPO Prediction (%) |
| ZCSE9\_gp32 | 100 | | ZCSE9\_gp20 | 0 | | ZCSE9\_gp45 | 0 |
| ZCSE9\_gp19 | 75 | | ZCSE9\_gp21 | 0 | | ZCSE9\_gp46 | 0 |
| ZCSE9\_gp31 | 53 | | ZCSE9\_gp22 | 0 | | ZCSE9\_gp47 | 0 |
| ZCSE9\_gp2 | 3 | | ZCSE9\_gp24 | 0 | | ZCSE9\_gp48 | 0 |
| ZCSE9\_gp3 | 1 | | ZCSE9\_gp25 | 0 | | ZCSE9\_gp49 | 0 |
| ZCSE9\_gp15 | 1 | | ZCSE9\_gp26 | 0 | | ZCSE9\_gp50 | 0 |
| ZCSE9\_gp23 | 1 | | ZCSE9\_gp27 | 0 | | ZCSE9\_gp51 | 0 |
| ZCSE9\_gp1 | 0 | | ZCSE9\_gp28 | 0 | | ZCSE9\_gp52 | 0 |
| ZCSE9\_gp4 | 0 | | ZCSE9\_gp29 | 0 | | ZCSE9\_gp53 | 0 |
| ZCSE9\_gp5 | 0 | | ZCSE9\_gp30 | 0 | | ZCSE9\_gp54 | 0 |
| ZCSE9\_gp6 | 0 | | ZCSE9\_gp33 | 0 | | ZCSE9\_gp55 | 0 |
| ZCSE9\_gp7 | 0 | | ZCSE9\_gp34 | 0 | | ZCSE9\_gp56 | 0 |
| ZCSE9\_gp8 | 0 | | ZCSE9\_gp35 | 0 | | ZCSE9\_gp57 | 0 |
| ZCSE9\_gp9 | 0 | | ZCSE9\_gp36 | 0 | | ZCSE9\_gp58 | 0 |
| ZCSE9\_gp10 | 0 | | ZCSE9\_gp37 | 0 | | ZCSE9\_gp59 | 0 |
| ZCSE9\_gp11 | 0 | | ZCSE9\_gp38 | 0 | | ZCSE9\_gp60 | 0 |
| ZCSE9\_gp12 | 0 | | ZCSE9\_gp39 | 0 | | ZCSE9\_gp61 | 0 |
| ZCSE9\_gp13 | 0 | | ZCSE9\_gp40 | 0 | | ZCSE9\_gp62 | 0 |
| ZCSE9\_gp14 | 0 | | ZCSE9\_gp41 | 0 | | ZCSE9\_gp63 | 0 |
| ZCSE9\_gp16 | 0 | | ZCSE9\_gp42 | 0 | | ZCSE9\_gp64 | 0 |
| ZCSE9\_gp17 | 0 | | ZCSE9\_gp43 | 0 | | ZCSE9\_gp65 | 0 |
| ZCSE9\_gp18 | 0 | | ZCSE9\_gp44 | 0 | | | |
Supplementary Table S2. Phage Depolymerase Finder Results [ANN model]

## Slide 7
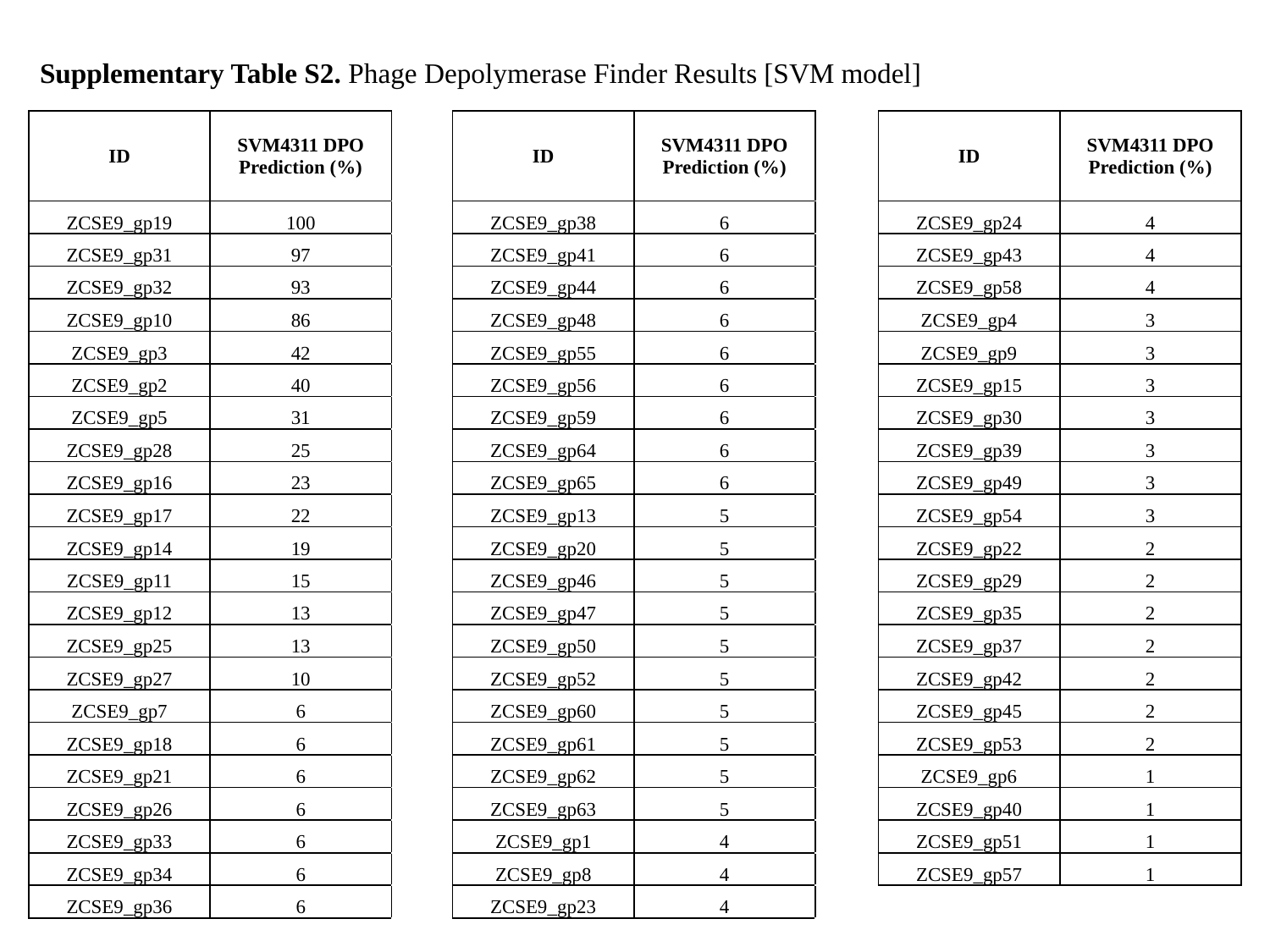

| | | | | | | | |
| --- | --- | --- | --- | --- | --- | --- | --- |
| ID | SVM4311 DPO Prediction (%) | | ID | SVM4311 DPO Prediction (%) | | ID | SVM4311 DPO Prediction (%) |
| ZCSE9\_gp19 | 100 | | ZCSE9\_gp38 | 6 | | ZCSE9\_gp24 | 4 |
| ZCSE9\_gp31 | 97 | | ZCSE9\_gp41 | 6 | | ZCSE9\_gp43 | 4 |
| ZCSE9\_gp32 | 93 | | ZCSE9\_gp44 | 6 | | ZCSE9\_gp58 | 4 |
| ZCSE9\_gp10 | 86 | | ZCSE9\_gp48 | 6 | | ZCSE9\_gp4 | 3 |
| ZCSE9\_gp3 | 42 | | ZCSE9\_gp55 | 6 | | ZCSE9\_gp9 | 3 |
| ZCSE9\_gp2 | 40 | | ZCSE9\_gp56 | 6 | | ZCSE9\_gp15 | 3 |
| ZCSE9\_gp5 | 31 | | ZCSE9\_gp59 | 6 | | ZCSE9\_gp30 | 3 |
| ZCSE9\_gp28 | 25 | | ZCSE9\_gp64 | 6 | | ZCSE9\_gp39 | 3 |
| ZCSE9\_gp16 | 23 | | ZCSE9\_gp65 | 6 | | ZCSE9\_gp49 | 3 |
| ZCSE9\_gp17 | 22 | | ZCSE9\_gp13 | 5 | | ZCSE9\_gp54 | 3 |
| ZCSE9\_gp14 | 19 | | ZCSE9\_gp20 | 5 | | ZCSE9\_gp22 | 2 |
| ZCSE9\_gp11 | 15 | | ZCSE9\_gp46 | 5 | | ZCSE9\_gp29 | 2 |
| ZCSE9\_gp12 | 13 | | ZCSE9\_gp47 | 5 | | ZCSE9\_gp35 | 2 |
| ZCSE9\_gp25 | 13 | | ZCSE9\_gp50 | 5 | | ZCSE9\_gp37 | 2 |
| ZCSE9\_gp27 | 10 | | ZCSE9\_gp52 | 5 | | ZCSE9\_gp42 | 2 |
| ZCSE9\_gp7 | 6 | | ZCSE9\_gp60 | 5 | | ZCSE9\_gp45 | 2 |
| ZCSE9\_gp18 | 6 | | ZCSE9\_gp61 | 5 | | ZCSE9\_gp53 | 2 |
| ZCSE9\_gp21 | 6 | | ZCSE9\_gp62 | 5 | | ZCSE9\_gp6 | 1 |
| ZCSE9\_gp26 | 6 | | ZCSE9\_gp63 | 5 | | ZCSE9\_gp40 | 1 |
| ZCSE9\_gp33 | 6 | | ZCSE9\_gp1 | 4 | | ZCSE9\_gp51 | 1 |
| ZCSE9\_gp34 | 6 | | ZCSE9\_gp8 | 4 | | ZCSE9\_gp57 | 1 |
| ZCSE9\_gp36 | 6 | | ZCSE9\_gp23 | 4 | | | |
Supplementary Table S2. Phage Depolymerase Finder Results [SVM model]

## Slide 8
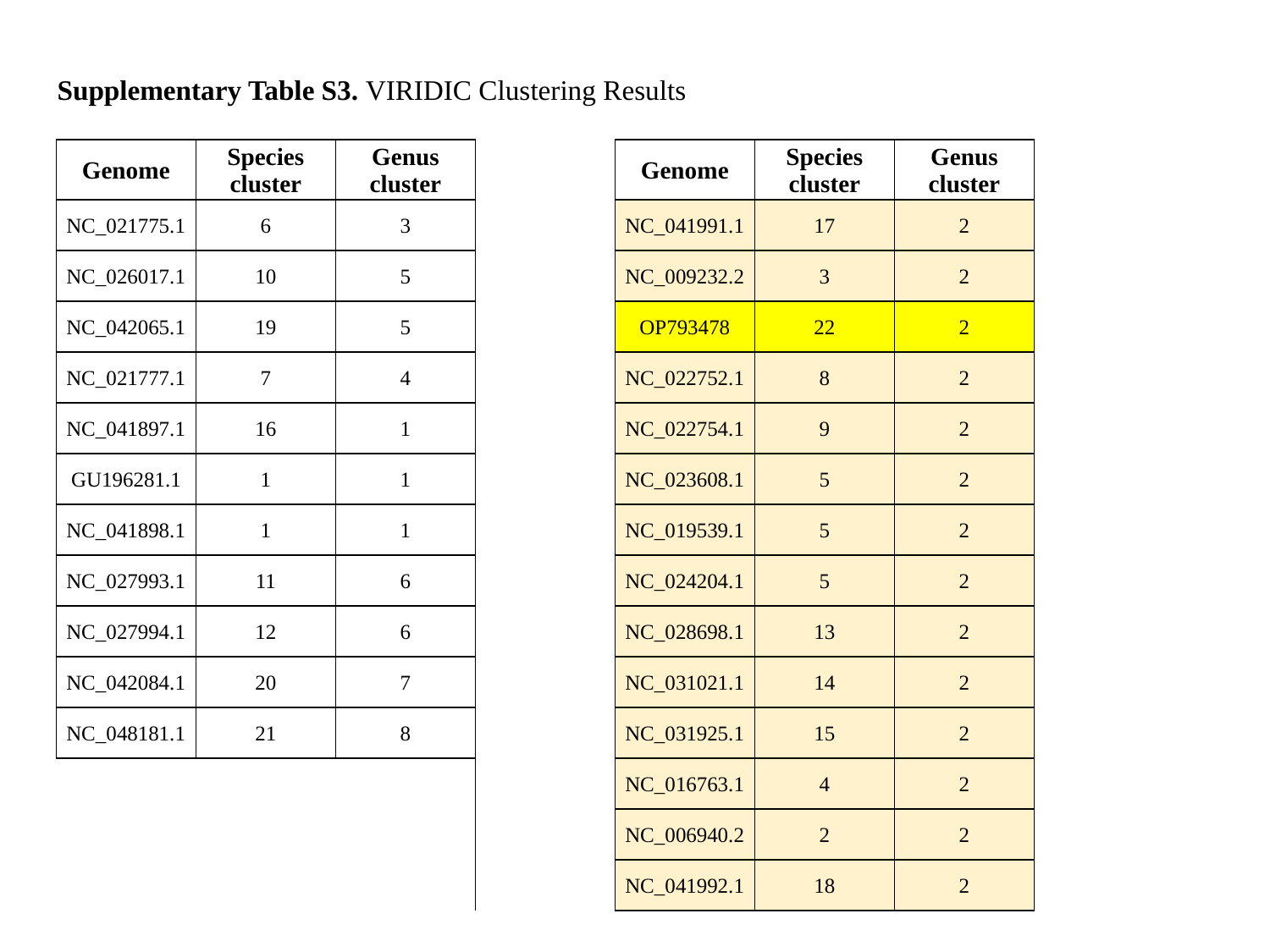

| Supplementary Table S3. VIRIDIC Clustering Results | | | | | | |
| --- | --- | --- | --- | --- | --- | --- |
| Genome | Species cluster | Genus cluster | | Genome | Species cluster | Genus cluster |
| NC\_021775.1 | 6 | 3 | | NC\_041991.1 | 17 | 2 |
| NC\_026017.1 | 10 | 5 | | NC\_009232.2 | 3 | 2 |
| NC\_042065.1 | 19 | 5 | | OP793478 | 22 | 2 |
| NC\_021777.1 | 7 | 4 | | NC\_022752.1 | 8 | 2 |
| NC\_041897.1 | 16 | 1 | | NC\_022754.1 | 9 | 2 |
| GU196281.1 | 1 | 1 | | NC\_023608.1 | 5 | 2 |
| NC\_041898.1 | 1 | 1 | | NC\_019539.1 | 5 | 2 |
| NC\_027993.1 | 11 | 6 | | NC\_024204.1 | 5 | 2 |
| NC\_027994.1 | 12 | 6 | | NC\_028698.1 | 13 | 2 |
| NC\_042084.1 | 20 | 7 | | NC\_031021.1 | 14 | 2 |
| NC\_048181.1 | 21 | 8 | | NC\_031925.1 | 15 | 2 |
| | | | | NC\_016763.1 | 4 | 2 |
| | | | | NC\_006940.2 | 2 | 2 |
| | | | | NC\_041992.1 | 18 | 2 |
